# A lightweight model for efficient identification of plant diseases and pests based on deep learning

**DOI:** 10.3389/fpls.2023.1227011

**Published:** 2023-07-14

**Authors:** Hongliang Guan, Chen Fu, Guangyuan Zhang, Kefeng Li, Peng Wang, Zhenfang Zhu

**Affiliations:** School of Information Science and Electrical Engineering, Shandong Jiaotong University, Jinan, China

**Keywords:** plant diseases and pests, deep learning, lightweight model, dynamic decay strategy, transfer learning

## Abstract

Plant diseases and pests have always been major contributors to losses that occur in agriculture. Currently, the use of deep learning-based convolutional neural network models allows for the accurate identification of different types of plant diseases and pests. To enable more efficient identification of plant diseases and pests, we design a novel network architecture called Dise-Efficient based on the EfficientNetV2 model. Our experiments demonstrate that training this model using a dynamic learning rate decay strategy can improve the accuracy of plant disease and pest identification. Furthermore, to improve the model’s generalization ability, transfer learning is incorporated into the training process. Experimental results indicate that the Dise-Efficient model boasts a compact size of 13.3 MB. After being trained using the dynamic learning rate decay strategy, the model achieves an accuracy of 99.80% on the Plant Village plant disease and pest dataset. Moreover, through transfer learning on the IP102 dataset, which represents real-world environmental conditions, the Dise-Efficient model achieves a recognition accuracy of 64.40% for plant disease and pest identification. In light of these results, the proposed Dise-Efficient model holds great potential as a valuable reference for the deployment of automatic plant disease and pest identification applications on mobile and embedded devices in the future.

## 1 Introduction

Plant diseases and pests can severely disrupt the normal growth and development of crops, leading to reduced crop yields and negatively impacting farmers’ income. Moreover, they can have severe implications for the supply of grains and agricultural products in the market, potentially resulting in a significant food crisis. Prioritizing the prevention and control of plant diseases and pests is essential in agricultural production, as effective management of these issues holds significant importance for ensuring food security, improving farmers’ income, and promoting sustainable agricultural development (Elnahal et al., 2022; Sehrawat et al., 2022).

Plant diseases and pests arise from a combination of environmental factors and pathogen invasion. Pathogens, which include fungi, bacteria, and viruses, are the fundamental cause of plant diseases. They can enter plant organisms through different transmission pathways, leading to the development of plant diseases and pests (Barragán-Fonseca et al., 2022). Environmental changes are also a critical factor in the onset and spread of plant diseases and pests (Canassa et al., 2020). Most plant diseases and pests exhibit distinct characteristics depending on the disease type, and accurately identifying the disease type based on these characteristics is crucial in effectively preventing and controlling plant diseases and pests.

In the past, people relied on visual observation of plant leaves and fruits to determine the presence of plant diseases and pests. They identified the type of plant disease based on the distinctive features exhibited by affected plants. However, this manual identification method heavily relied on individual experience, resulting in high labor costs and low efficiency. Subsequently, with the advancement of computer technology, machine learning techniques were introduced to aid in the identification of plant diseases and pests. At first, machine learning utilized computer vision to analyze the morphological changes in diseased leaves or fruits and extract the pathological features of plant diseases. The computer then made predictions about the disease type based on the obtained features. However, machine learning-based methods for automated plant disease and pest identification faced limitations in terms of accuracy and generalizability. The use of rule-based image processing techniques to extract disease features led to sensitivity to image quality, as image noise could greatly affect the final results (Behmann et al., 2015; Wani et al., 2022).

In recent years, deep learning has made significant breakthroughs and has taken the forefront as become a research direction in computer vision, particularly in the field of agriculture. In this context, the use of deep learning for plant disease and pest type identification has emerged as an important application and research area (Liu and Wang, 2021). Currently, deep learning-based models for plant disease and pest identification are exhibiting a trend toward increased accuracy, smaller model sizes, faster training speeds, and stronger transferability. In response to this trend, this paper proposes a lightweight model for the efficient identification of plant diseases and pests based on deep learning, called the Dise-Efficient model.

The main contributions of this study are as follows:

1. Proposing the Dise-Efficient model, a novel deep learning-based model for efficient and accurate identification of plant diseases and pests.
2. Demonstrating how the number of convolutional layers and the size of the convolution kernel affect the accuracy of the Dise-Efficient model in identifying plant diseases and pests.
3. Training the Dise-Efficient model using the dynamic learning rate decay strategy and experimentally demonstrating that this strategy can significantly improve the accuracy of the model.
4. Excrementally validating the Dise-Efficient model has a good transfer learning ability.

## 2 Related work

The advancement of deep learning technology has led to rapid progress in the field of plant pest detection. The research on the automatic identification of plant pests and diseases has witnessed an evolution of convolutional neural network (CNN) models from small to large, resulting in continual improvement in accuracy rates. More recently, however, there has been a shift toward developing more lightweight models that maintain high accuracy rates while having smaller model sizes.

### 2.1 Convolutional neural network models

Following the proposal of the AlexNet model by Krizhevsky et al. (Krizhevsky et al., 2017), there has been rapid development of CNNs in the field of computer image recognition. Subsequently, CNN models began to be applied to the agricultural field. According to the experimental results presented by Mohanty et al. (Mohanty et al., 2016), the AlexNet model can achieve an accuracy rate of 99.28% in identifying plant diseases and pests on the Plant Village public dataset. This indicates the effectiveness of CNN models in identifying plant diseases and pests. He et al. (He et al., 2016) proposed a ResNet model, which involved adding an increased number of convolutional layers to a CNN model, as an improvement to the accuracy of image recognition. Following this, researchers have used the concept of the ResNet model to design CNN models with deep convolutional layers across various image recognition applications. The aim is to improve the accuracy of CNN models in identifying different image types. Fuentes et al. (Fuentes et al., 2019) used ResNet50 as the feature extractor in the SSD target detection framework to identify potato diseases, resulting in an accuracy rate of 85.98%. Similarly, Kumar et al. (Kumar et al., 2020) implemented the ResNet34 model to identify 14 different crop diseases on the Plant Village dataset, with a high accuracy rate of 99.40%.

As CNN models achieved high accuracy rates, researchers started exploring the issue of making the model lightweight. The emergence of lightweight CNN models such as MobileNet and EfficientNet has led the research on plant disease image recognition towards the development of lightweight CNN models (Howard et al., 2019; Tan and Le, 2019). Lightweight CNN models usually use depthwise (DW) separable convolution (DW) to replace ordinary convolution, reducing model and parameter size. However, this approach may result in a decline in recognition accuracy. To deal with this problem, a common approach is to add a squeeze and excitation (SE) block (Hu et al., 2018) to lightweight models to improve their accuracy in identifying image types. Many lightweight CNN model structures, such as the EfficientNetV2 model (Tan and Le, 2021), have been proposed based on this concept. SE blocks are often added to ensure the accuracy of the model. Kamal et al. (Kamal et al., 2019) used the original MobileNet model to train their proposed model on the Plant Village dataset, achieving an accuracy rate of 98.65%. However, when compared to traditional CNN models such as AlaxNet and VGG, there was a decrease in the accuracy rate by approximately 1%. Chen et al. (Chen et al., 2021) embedded the SE block into MobileNet and trained it on the Plant Village dataset, achieving an accuracy rate of 99.78%, which surpassed those obtained by many traditional CNN models trained on this dataset for plant disease type identification.

### 2.2 Learning strategies

Initially, researchers used a fixed learning rate to train the CNN model, which caused the accuracy of the model to be heavily dependent on the learning rate parameter. Later, many researchers improved the training speed and identification accuracy of the model by proposing strategies for adjusting the learning rate parameter. These strategies can be categorized into two main groups: adaptive learning rate and learning rate decay. Among them, the Adam optimizer, which utilizes an adaptive learning rate strategy, is widely used in deep learning and is known for its effectiveness. Loshchilov et al. (Loshchilov and Hutter, 2017) proposed a cosine processing strategy to dynamically adjust the learning rate. He et al. (He et al., 2019) applied the cosine learning rate decay strategy to train the ResNet50 model, resulting in an improvement of approximately 2% in model accuracy.

Inspired by the successful application of dynamic learning rate, this paper applies the cosine-type progressive learning rate decay strategy to the Dise-Efficient model to improve the model’s accuracy in identifying plant diseases and pests. Formula (1) outlines the dynamic learning rate decay strategy proposed in this paper:


(1) $$lr=\left(1+\cos\frac{\pi x}{n}\right)\cdot \left(1-lrf\right)+lrf$$

where lr represents the learning rate of the next round; lrf represents the learning rate of the last round; x represents the learning rate of the current round; *n* represents the maximum number of iterations.

### 2.3 Transfer learning

Recent CNN models have shown high accuracy rates of over 95% on the Plant Village plant disease dataset (Ahmad et al., 2022). However, the performance of these CNN models on the IP102 large-scale plant pest dataset is lower than expected, with traditional CNN models achieving an accuracy rate of around 50% (Ren et al., 2019; Wu et al., 2019; Nurfauzi et al., 2023). Despite the improvements made to the CNN models, their accuracy on this dataset is only slightly over 60% (Nanni et al., 2020). This can be explained by the fact that the IP102 dataset is a plant pest dataset that reflects the actual environment, with images possessing more complex backgrounds and fewer samples for each pest category. Therefore, conducting deep learning model training utilizing transfer learning is an effective solution to address the issue of limited data samples for certain pest categories in the IP102 dataset.

Transfer learning involves transferring the knowledge or patterns learned from existing labeled training data to improve learning in a new target field (Weiss et al., 2016). Incorporating transfer learning in the deep learning model training process not only accelerates the model training process but also facilitates the acquisition of a more accurate deep learning model through the fine-tuning of the pre-trained model (Zhu et al., 2023). In current research on plant disease and pest identification, many researchers have applied transfer learning to CNN models to improve both the training speed of the model and the accuracy of identification (Thenmozhi and Reddy, 2019; Liu et al., 2022).

## 3 Experiments

### 3.1 Dataset and environment

Plant Village is a public plant disease dataset (Hughes and Salathé, 2015), containing 54,303 images of healthy or diseased leaves categorized into 38 different groups from 9 crop species. Researchers often utilize this dataset in studies related to the identification of plant diseases and pests, as well as for developing models aimed at identifying various types of plant pests.

IP102 is a large-scale dataset developed for identifying pests (Wu et al., 2019), comprising more than 75,000 images categorized into 102 types, exhibiting a natural long-tail distribution. IP102 has a hierarchical taxonomy that groups pests that primarily affect one particular agricultural product into the same upper category. This dataset is often used in research aimed at identifying plant pests and is implemented in this study as a training dataset for the plant pest identification model.

The Mini-ImageNet dataset (Satorras and Estrach, 2018) comprises 100 common categories selected from the ImageNet dataset, with each category containing 600 images and a total of 60,000 images. Given that this dataset is often used in the pre-training of small sample learning models, it is employed as the dataset for the pre-trained model in the present study.

Before it is applied for model training, the dataset must be split into different sets. In this study, we divided the Plant Village and IP102 datasets into a training set, a validation set, and a test set at a ratio of 3:1:1. Meanwhile, the Mini-ImageNet dataset was used for the pre-training model, so it was divided into a training set and a validation set at a ratio of 4:1. **Table 1** shows the number of images present in the different sets of each divided dataset.

**Table 1:** Number of images in different sets of each divided dataset.

Dataset	Training set/sheet	Validation set/sheet	Test set/sheet	Total/sheet
Plant Village	36,892	12,297	12,297	61,486
IP102	45,132	15,043	15,043	75,218
Mini-ImageNet	48,000	12,000	/	60,000

During the training phase of this experimental model, we employed the Tencent Cloud GN7-8-core 32G cloud server that supports GPU computing tasks. The GPU model used was Nvidia Tesla T4, featuring 16 GB video memory and 32 GB internal memory, and the operating system was Ubuntu Server 20.04 LTS 64-bit with a Cuda version of 11.2.

### 3.2 Model

**Figure 1:**
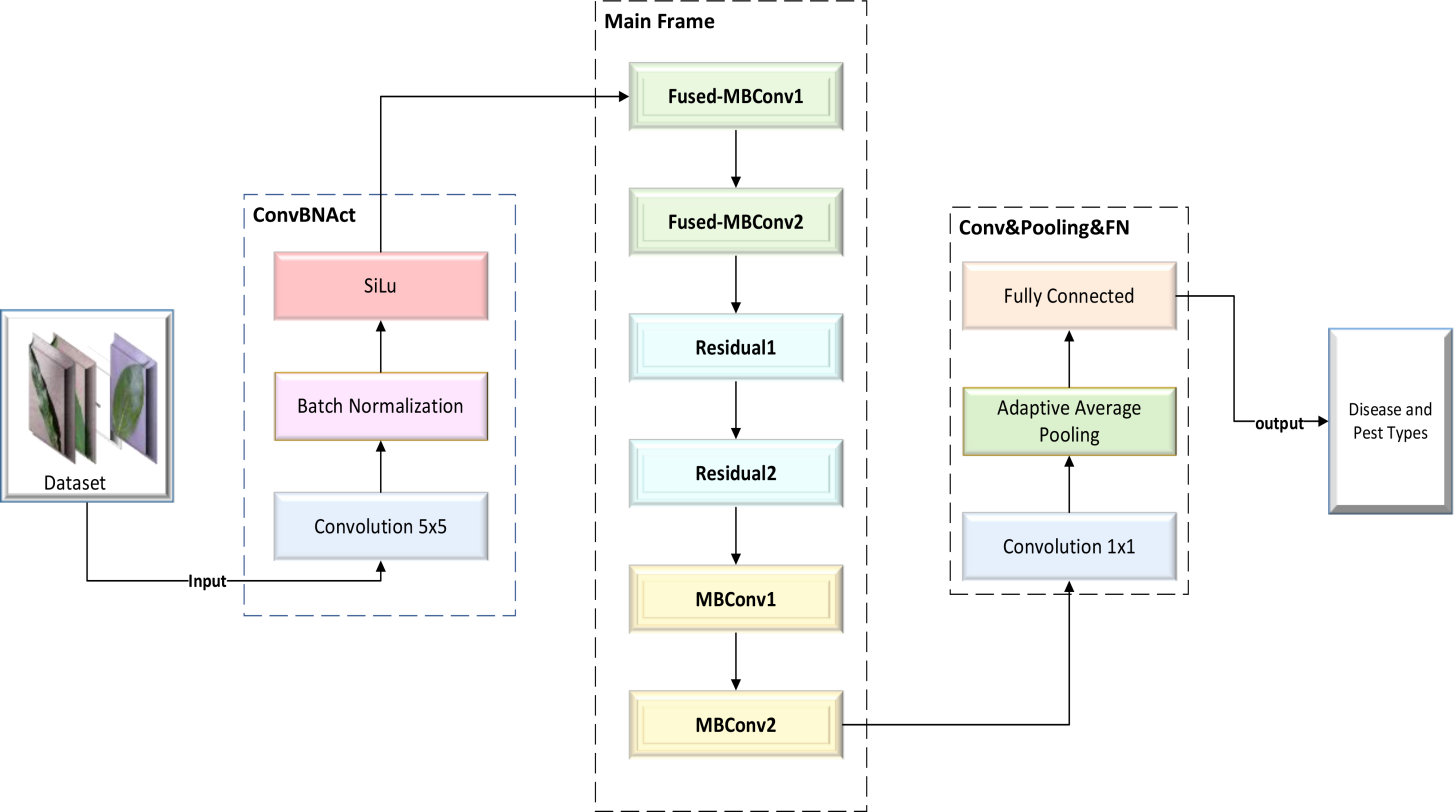
Framework of the Dise-Efficient model.

Drawing upon our previous research experience, we thoroughly studied the structures and principles of the classic ResNet model and the lightweight EfficientNetV2 model. After careful consideration, we decided to use the residual block of ResNet to replace a portion of the MBConv block and Fused-MBConv block in the EfficientNetV2 model. Finally, we managed to design a lightweight CNN network model that can efficiently identify various types of plant diseases and pests: the Dise-Efficient model. The framework of this model is shown in **Figure 1**.

**Figure 2:**
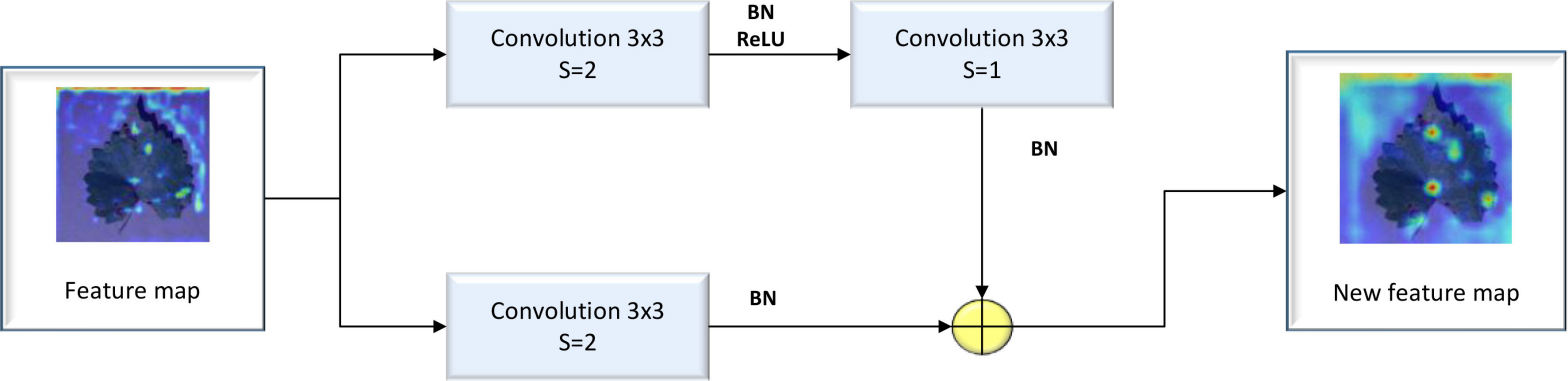
Structure of the Residual block.

The Residual block is regarded as the basic residual block of ResNet18. It features three convolution kernels with a size of 3×3, along with a shortcut connection. The residual block can add the original feature map to the feature map resulting from the convolution process to obtain a new feature map. Because the image feature distribution of diseased crop leaves is relatively simple, issues of gradient explosion and gradient disappearance may arise due to the continuous deepening of the convolutional layer. These problems can be addressed by incorporating a Residual layer, which allows for the extraction of deep features from diseased crop leaf images. A detailed illustration of the Residual block’s structure is provided in **Figure 2**.

**Figure 3:**
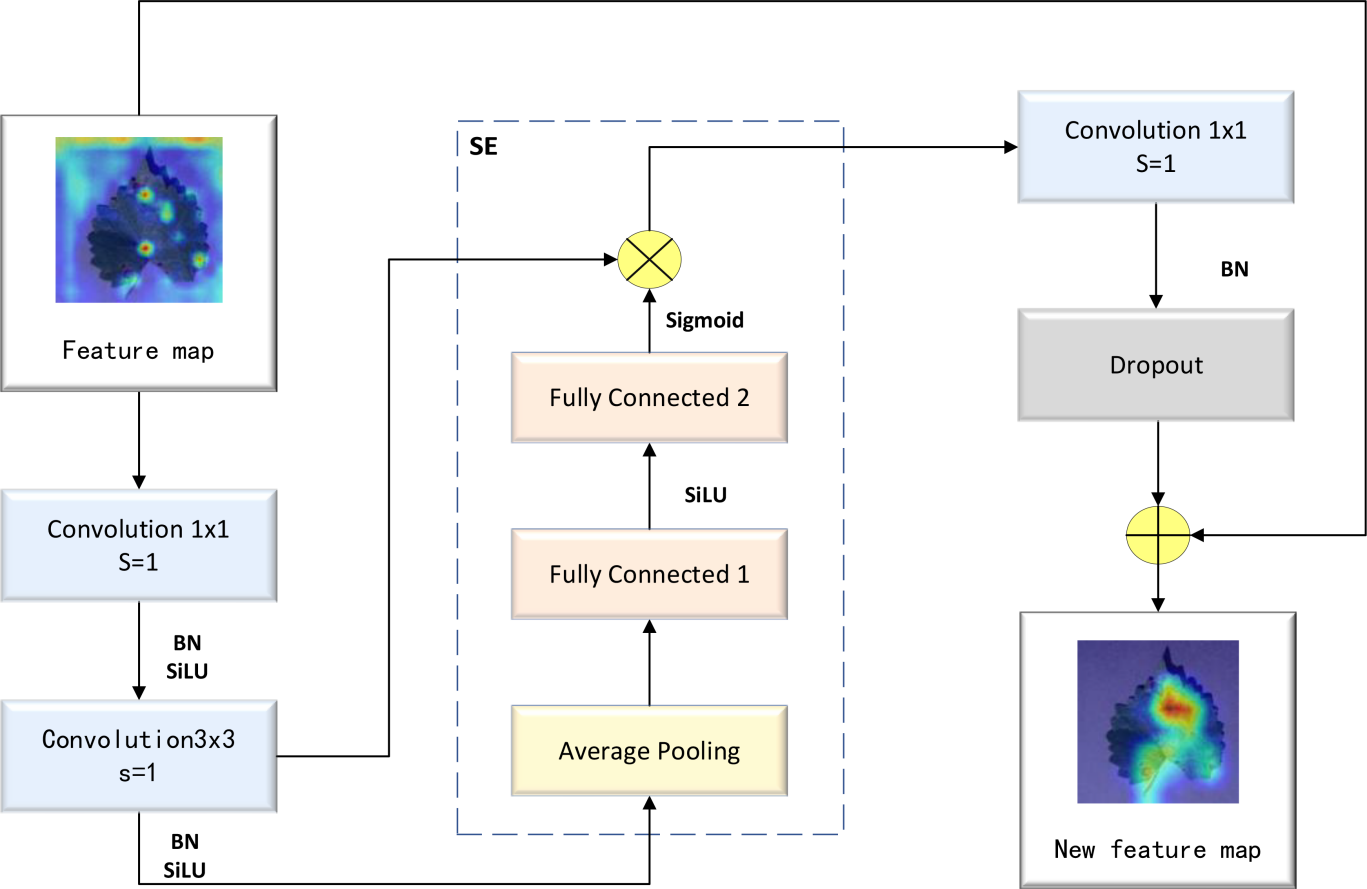
Structure of the MBConv block.

The MBConv block represents an improvement based on the residual block. First, the ordinary convolution operation was replaced with a DW separable convolution operation. This involved adding two convolution kernels with a size of 1×1 into the residual structure, thereby realizing a DW separable convolution operation. Subsequently, a compression and excitation layer was added to enhance the self-attention mechanism of the model and mitigate the reduction in accuracy caused by a decrease in the number of parameters. As a result of these adjustments, the prediction accuracy of the model was improved. A detailed illustration of the MBConv block’s structure is provided in **Figure 3**.

**Figure 4:**
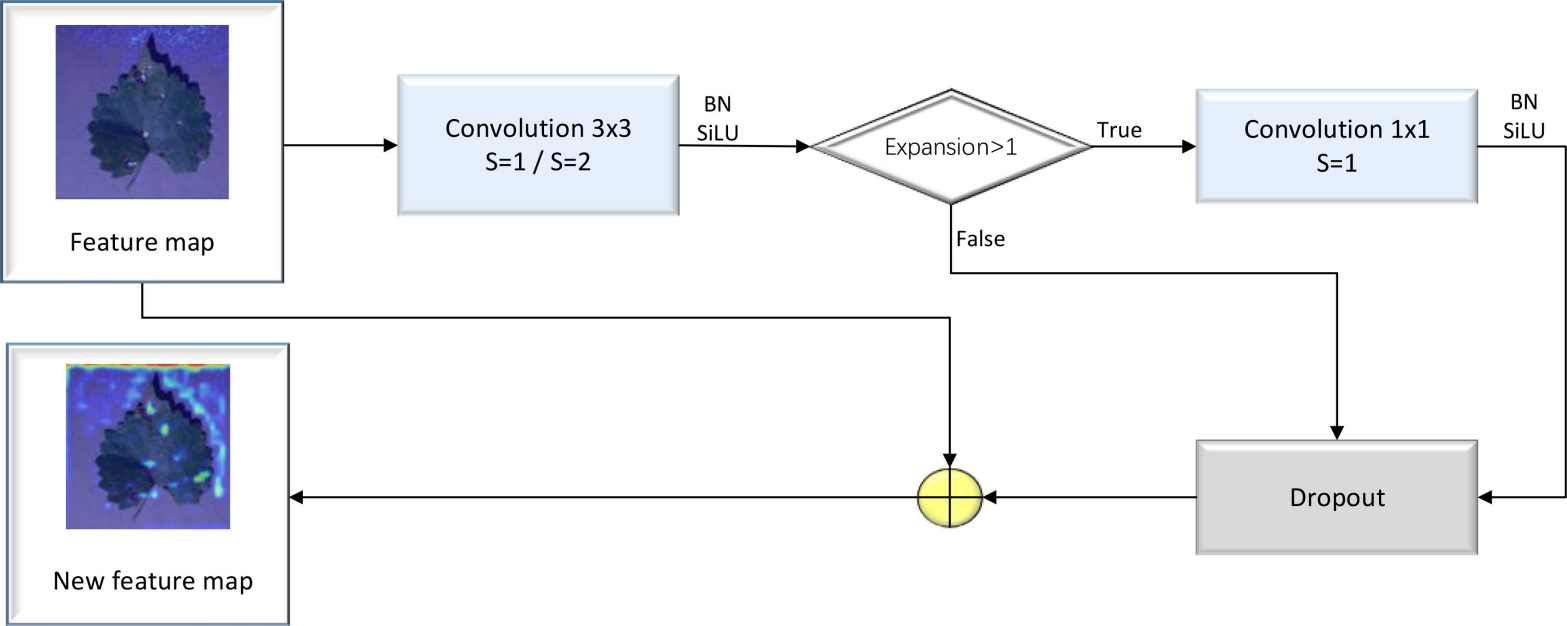
Structure of the Fused-MBCov block.

The Fused-MBConv block is a modified version of the MBConv block, which involves removing the first convolutional layer for dimensionality increment and the data squeezing and excitation layer in the MBConv module. The block was used to determine whether DW separable volumes are to be performed based on the expansion coefficient point-by-point operations of the product. A detailed illustration of the Fused-MBConv block’s structure is provided in **Figure 4**.

### 3.3 Experimental design

#### 3.3.1 Experimental comparison of convolutional layers of different models

To verify the effect of different convolutional layers on the accuracy of the Dise-Efficient model in identifying plant disease types, we designed a baseline model called Dise-Efficient-B0-N, also referred to as B0-N. In this baseline model, each convolutional layer consists of two layers. In addition, we developed the B0-S model, which is smaller than the B0-N model, and the B0-L model, which is larger than the B0-N model.

In the experiment, we trained the B0-N, B0-S, and B0-L models on the Plant Village dataset. After the training, we compared the accuracy of the three models in identifying plant disease types. The main parameters of the three models are presented in **Table 2**.

**Table 2:** Parameters of the B0-N, B0-S, and B0-L models.

Block	B0-N Layers	B0-S Layers	B0-L Layers	Stride	Number of convolution kernels	Dropout	Expansion
ConvBNAct	1	1	1	2	32	0	–
Fused-MBConv1	2	1	3	1	32	0	1
Fused-MBConv2	2	1	3	2	64	0	4
Residual1	2	1	3	2	64	0	–
Residual2	2	1	3	2	128	0	–
MBConv1	2	1	3	1	160	0.25	6
MBConv2	2	1	3	2	256	0.25	6

#### 3.3.2 Experimental comparison of different learning strategies

**Figure 5:**
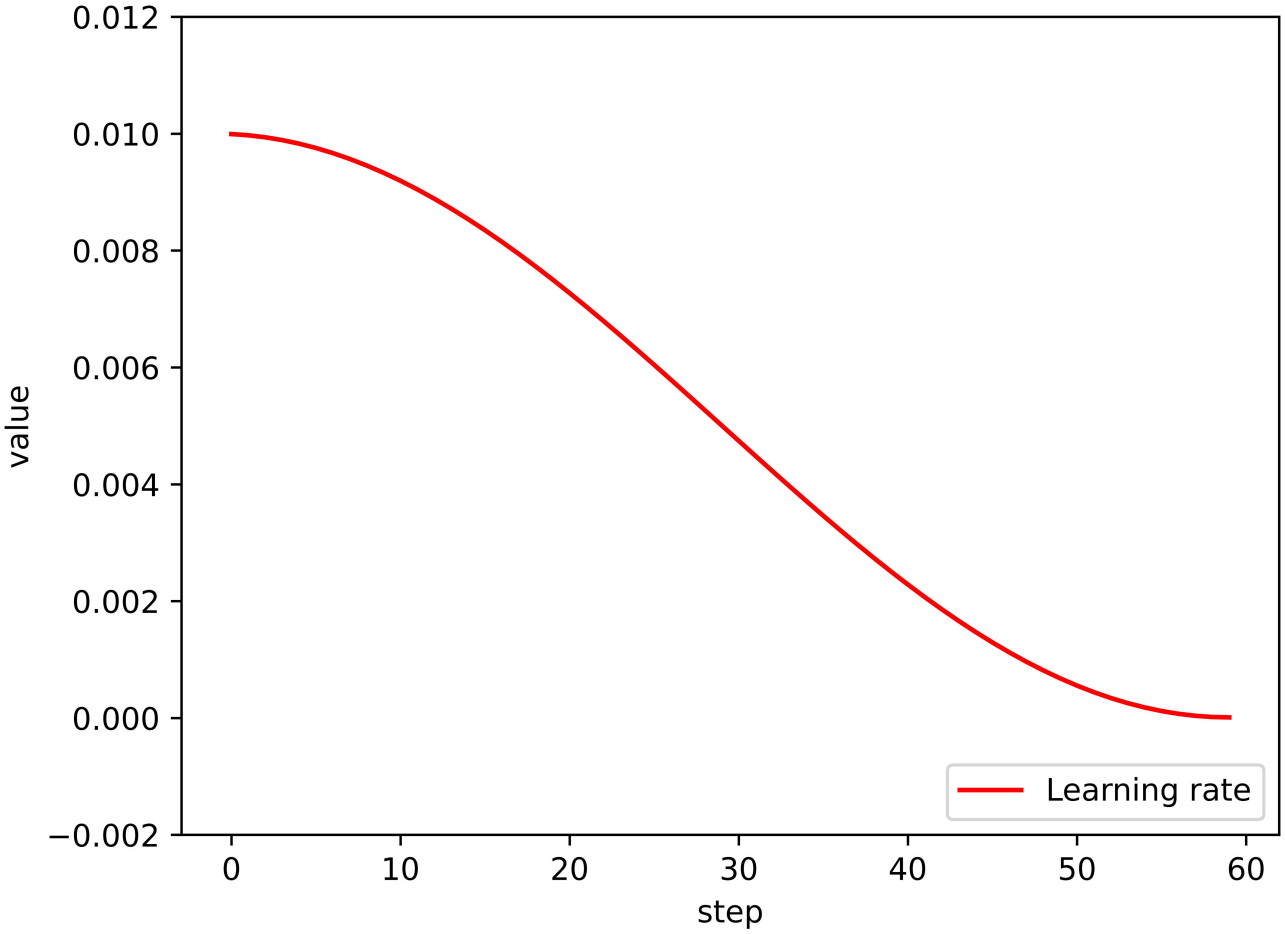
Learning rate decay result.

The learning strategy designed in this study is comprised of a stochastic gradient descent (SGD) optimizer, which utilizes momentum to improve the model training process. Additionally, we implemented a cosine dynamic decay strategy for the learning rate, which started at 0.01 and decayed in a cosine manner as the number of training rounds increased. Formula (1) illustrates the dynamic decay strategy for the learning rate, with the final learning rate being 0.001. The learning rate decay result is depicted in the form of a curve in **Figure 5**.

Generally, the Adam optimizer provides better optimization performance for model training than the SGD optimizer combined with the momentum learning strategy. However, our experiments revealed that the model generally achieved higher accuracy in identifying disease types when the SGD optimizer was implemented in combination with the cosine dynamic decay strategy, as designed in this paper, compared to when the Adam optimizer was used.

To verify whether the cosine dynamic decay learning strategy can improve the accuracy of the automatic plant disease and pest identification model, we conducted experiments on the Plant Village dataset, using the B0-N, B0-S, and B0-L models for comparative analysis. In experimental group 1, we implemented the Adam optimizer commonly used in CNN model training, while setting the learning rate parameter to a fixed value of 0.001. In experimental group 2, we utilized the SGD optimizer with a fixed learning rate. In the control group, we employed the SGD optimizer with a cosine dynamic decay strategy that gradually reduced the learning rate from 0.01 to 0.001 based on formula (1). The specific experimental parameters are listed in **Table 3**.

**Table 3:** Experimental conditions and parameters.

	Experimental group 1	Experimental group 2	Control group
Learning strategy	Fixed learning rate	Fixed learning rate	Cosine dynamic attenuation
Optimizer	Adam	SGD	SGD
Momentum	/	0.9	0.9
Initial learning rate (lr)	0.001	0.001	0.01
Final learning rate (lrf)	0.001	0.001	0.001
Epochs	60	60	60
Batch size	64	64	64

#### 3.3.3 Experimental comparison of convolution kernel sizes of different models

Generally, smaller convolution kernels tend to capture finer-grained features, while larger ones are better suited for capturing more macroscopic features (Szegedy et al., 2015). Therefore, by changing the size of the convolution kernel and observing how the accuracy of the model accordingly, we can understand the effect of different feature scales on the performance of the model. With this in mind, we changed the size of the module convolution kernel to investigate the effect of replacing a small convolution kernel with a large one on each module’s performance.

In this experiment, we constructed models from Dise-Efficient-B1 to Dise-Efficient-B7, all based on the Dise-Efficient-B0-N (abbreviated as B0) model. Specifically, the B1 to B7 models were designed with 5x5 large convolution kernels to replace the 3x3 small convolution kernels of different modules. **Table 4** shows the details of the convolution kernel replacements, and other parameters remain unchanged from the B0 model.

**Table 4:** Number of model layers and convolution kernel size.

Block	B0	B1	B2	B3	B4	B5	B6	B7
BNConvAct	3x3							
Fused-Conv1	3x3	5x5	3x3	3x3	5x5	5x5	3x3	5x5
Fused-Conv2	3x3	5x5	3x3	3x3	5x5	5x5	3x3	5x5
Residual1	3x3	3x3	5x5	3x3	5x5	3x3	5x5	5x5
Residual2	3x3	3x3	5x5	3x3	5x5	3x3	5x5	5x5
MBConv1	3x3	3x3	3x3	5x5	3x3	5x5	5x5	5x5
MBConv2	3x3	3x3	3x3	5x5	3x3	5x5	5x5	5x5

#### 3.3.4 Experimental comparison of transfer learning abilities of different models

**Figure 6:**
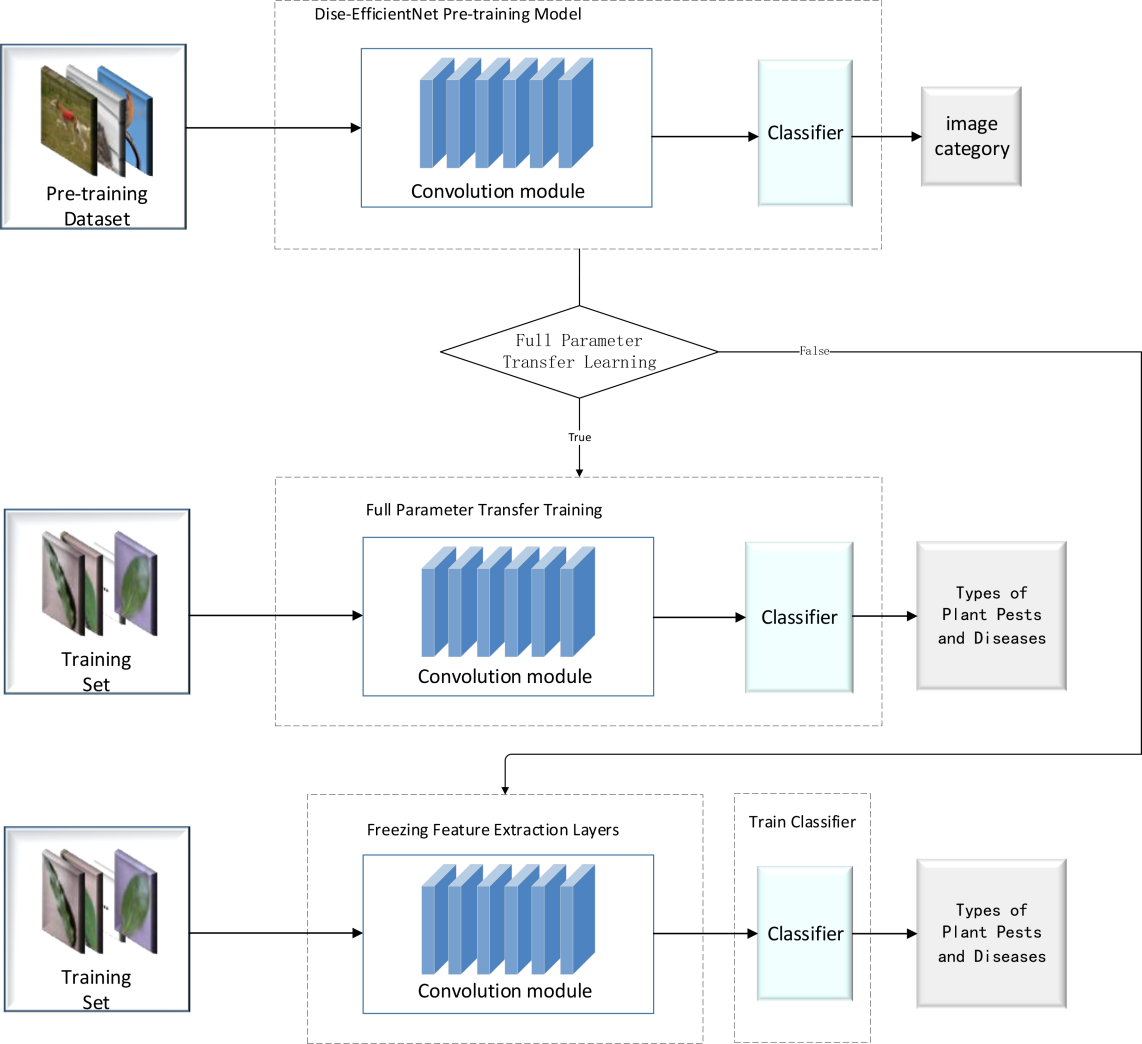
Flowchart of transfer learning.

The migration learning process consists of two phases: pre-training and migration learning. In the pre-training phase of this experiment, we used the cosine dynamic learning rate decay strategy designed in this study to train the B0 and B2 models, as shown in **Table 4**, on the Mini-ImageNet dataset, generating pre-training models for B0 and B2. Finally, the pre-trained model weights were uploaded in the IP02 dataset for use in the transfer learning process, as illustrated in **Figure 6**.

In this study, two transfer learning methods were used to compare the experimental results. The first one involved freezing the feature layer of the pre-trained model before performing transfer learning. The second one involved using the full set of parameters for direct transfer learning. Details of the specific experimental design are shown in **Table 5**.

**Table 5:** Experimental design of transfer learning.

Model name		Original model	Feature layer freezing	Full parameter transfer
B0	B2	**√**		
B0-Freeze-TF	B2-Freeze-TF		**√**	
B0-TF	B2-TF			**√**

## 4 Results and analysis

### 4.1 Validity of the model

To evaluate the performance of the proposed Dise-Efficient model in identifying plant pest types, we trained the baseline model Dise-Efficient-B0 on ​​the Plant Village dataset. This experiment was conducted under the experimental conditions and parameters for the experimental groups in **Table 3**. We compared the accuracy rate obtained by the final model on the test set with the accuracy rates of other CNN models used for agricultural pest detection. The comparison results are presented in **Table 6**.

**Table 6:** Comparison between Dise-Efficient and other plant disease and pest identification models.

Dataset	Research paper	Model name	Accuracy (%)	Dataset	Research paper	Model name	Accuracy (%)
Plant Village	Sladojevic et al., 2016	CaffeNet	98.21	IP102	Nurfauzi et al., 2023	EfficientNetV2-B0	51.00
	Gokulnath, 2021	LF-CNN	98.93		Ren et al., 2019	FR-ResNets	55.24
	Ganatra and Patel, 2020	Inception V4	98.30		Lin et al., 2023	GPA-Net	56.90
	Bedi and Gole, 2021	Models in the research	99.38		Nanni et al., 2020	**Models in the research**	**61.93**
	**Ours**	**Dise-Efficient-B0-N**	**99.71**		Ours	Dise-Efficient-B0	61.48

Bold values mean the line with the best model evaluation index.

From the results in **Table 6**, it can be seen that the Dise-Efficient-B0 model achieved the highest accuracy rate in identifying plant disease types on the Plant Village dataset, reaching 99.71%. The model delivered a 61.84% accuracy rate in identifying plant pest types on the IP102 dataset, which was only lower than the accuracy rate of a previously proposed model (Nanni et al., 2020). These findings demonstrated that the Dise-Efficient model has a strong ability in identifying various types of plant diseases and pests. Therefore, this model holds substantial research and practical value for the identification of plant diseases and pests.

### 4.2 Effect of the number of convolutional layers on model performance

To investigate the effect of the number of convolutional layers on the accuracy of plant disease and pest identification models, we experimentally implemented the B0-N, B0-S, and B0-L models presented in **Table 1** under the experimental conditions and parameters for the experimental groups in **Table 3**. Finally, we obtained the indexes of the models in the experimental groups, as shown in **Table 7**.

**Table 7:** Indexes of different models in the experimental groups.

Index	Dise-Efficient-B0-N	Dise-Efficient-B0-S	Dise-Efficient-B0-L
Accuracy/%	**99.71**	99.55	99.60
Model size/MB	13.30	**5.86**	20.80

Bold values mean the line with the best model evaluation index.

The above results indicate that the B0-N model is the most accurate in identifying plant disease types, achieving an accuracy rate of 99.71%. Furthermore, the B0-S model is the smallest in size, at only 5.86 MB, but delivers a 0.16% lower accuracy rate than the B0-N model. In contrast, the B0-L model has the largest size, measuring 20.80 MB.

Through an analysis of the above results, we found that the B0-S model has one less convolutional layer in each module when compared to B0-N, so the model size of B0-S is smaller than that of B0-N; the B0-L model has one more layer convolutional layer in each module when compared to B0-N, so the model size of B0-L is larger than that of B0-N. Hence, the number of convolutional layers will directly affect the model size – the more convolutional layers, the larger the model size.

### 4.3 Effect of dynamic learning strategy on model performance

Based on the experimental design in **Table 3**, we obtained the accuracy and model size of the Dise-Efficient model used for identifying plant disease types in experimental group 1, experimental group 2, and the control group on the Plant Village test set. The results are shown in **Table 8**.

**Table 8:** Model indexes for comparison of experimental results.

Index	B0-N	B0-S	B0-L
Accuracy for experimental group 1 (%)	99.71	99.55	99.60
Accuracy for experimental group 2 (%)	99.27	99.19	99.51
**Accuracy for control group (%)**	**99.81**	**99.77**	**99.82**

Bold values mean the line with the best model evaluation index.

We implemented the Adam optimizer with a fixed learning rate for experimental group 1 and the SGD optimizer with a fixed learning rate for experimental group 2. From **Table 8**, it can be seen that for the same model trained under a fixed learning rate strategy, using the Adam optimizer for training leads to higher accuracy rates compared to using the SGD optimizer. In the control group, we used the SGD optimizer in combination with the cosine dynamic learning decay strategy to train the model, resulting in a higher accuracy in identifying plant disease types than the model trained under the conditions and parameters for experimental group 1. It can be concluded that incorporating a cosine dynamic learning rate decay strategy into the model training process can improve the model’s accuracy in identifying plant diseases and pests.

### 4.4 Effect of convolution kernel size on model performance

The experiment was conducted based on the B0-N model (B0 for short), which had its convolution kernel replaced according to the design in **Table 3**, resulting in the creation of models B1 to B7. Models B0 to B7 were trained on the IP102 plant pest dataset utilizing the experimental conditions and parameters for the control group outlined in **Table 3**. The trained model’s accuracy and other indexes of these models are presented in **Table 9**.

**Table 9:** Indexes of Dise-Efficient-B0 to B7 models.

Model	Accuracy (%)	Increase in accuracy (%)	Model size (MB)	Increase in size (MB)
Dise-Efficient-B0	61.48	0	**13.3**	0
Dise-Efficient-B1	61.24	-0.24	15.0	+1.7
Dise-Efficient-B2	**61.84**	**+0.36**	16.1	+2.8
Dise-Efficient-B3	61.39	-0.09	13.9	+0.6
Dise-Efficient-B4	61.32	-0.16	17.5	+4.2
Dise-Efficient-B5	60.75	-0.73	15.3	+2.0
Dise-Efficient-B6	61.32	-0.16	16.4	+3.1
Dise-Efficient-B7	61.45	-0.03	17.8	**+4.5**

Bold values mean the line with the best model evaluation index.

Based on the abovementioned experimental findings, it is evident that replacing a small-sized ordinary convolution kernel with a larger one usually improves the accuracy of the Dise-Efficient model in identifying plant pest types. However, replacing a small-sized DW separable convolution kernel with a larger one negatively affects the model’s accuracy in identifying plant pest types.

It can be seen from **Figure 1** that the Residual block of the Dise-Efficient model is the only one utilizing a common convolution kernel, while the MBConv and Fused-MBConv blocks use a DW separable convolution kernel. From the convolution kernel sizes set for the different blocks of each model in **Table 4**, it can be seen that Dise-Efficient-B2 only replaces the small convolution kernel with a larger one in its Residual block. Consequently, this model experiences a substantial improvement in identifying plant pest types, which is evidenced by a peak accuracy rate of 61.84%. As for models B1 and B3, they only replace the DW convolution kernel in their Fused-MBConv and MBConv blocks. As a result, both of these models experience varying degrees of reductions in accuracy.

**Table 8** shows that the Dise-Efficient-B5 model delivers the lowest accuracy rate, likely due to its use of a larger DW convolution kernel in the place of a smaller one in its Fused-MBConv and MBConv blocks. This replacement caused the model’s accuracy in identifying plant pest types to experience the largest drop. Additionally, models B4, B6, and B7 all replace smaller DW convolution kernels with larger ones, leading to varying degrees of reductions in the accuracy in identifying plant pest types.

In terms of the number of parameters, a DW convolution kernel of the same specification has fewer parameters than the ordinary convolution kernel. Therefore, replacing the ordinary convolution kernel with a larger one will increase the size of the model compared to replacing a DW convolution kernel. As a result, as illustrated in **Table 8**, B2 experiences a more significant increase in model size when compared to B1 and B3. This can be explained by the fact that B2 replaces the ordinary convolution kernel with a larger one, while B1 and B3 replace simply DW convolution kernels. Similarly, model B4 only replaces DW convolution kernels in the Fused-MBConv and MBConv blocks without replacing the ordinary convolution kernel, leading to a smaller increase in model size compared to B4, B6, and B7.

Through the above analysis, it can be concluded that in the application of the Dise-Efficient model for identifying plant pests, replacing the convolution kernel in the Residual module with a larger one can improve the model’s accuracy in plant pest type identification, although this improvement comes at the cost of increased model size. In contrast, while replacing a smaller DW convolution kernel with a larger one only causes a small increase in model size, it results in a reduction in accuracy. Therefore, sacrificing a lightweight cost for greater accuracy improvement could be a meaningful research direction to explore.

### 4.5 Application of transfer learning

**Figure 7:**
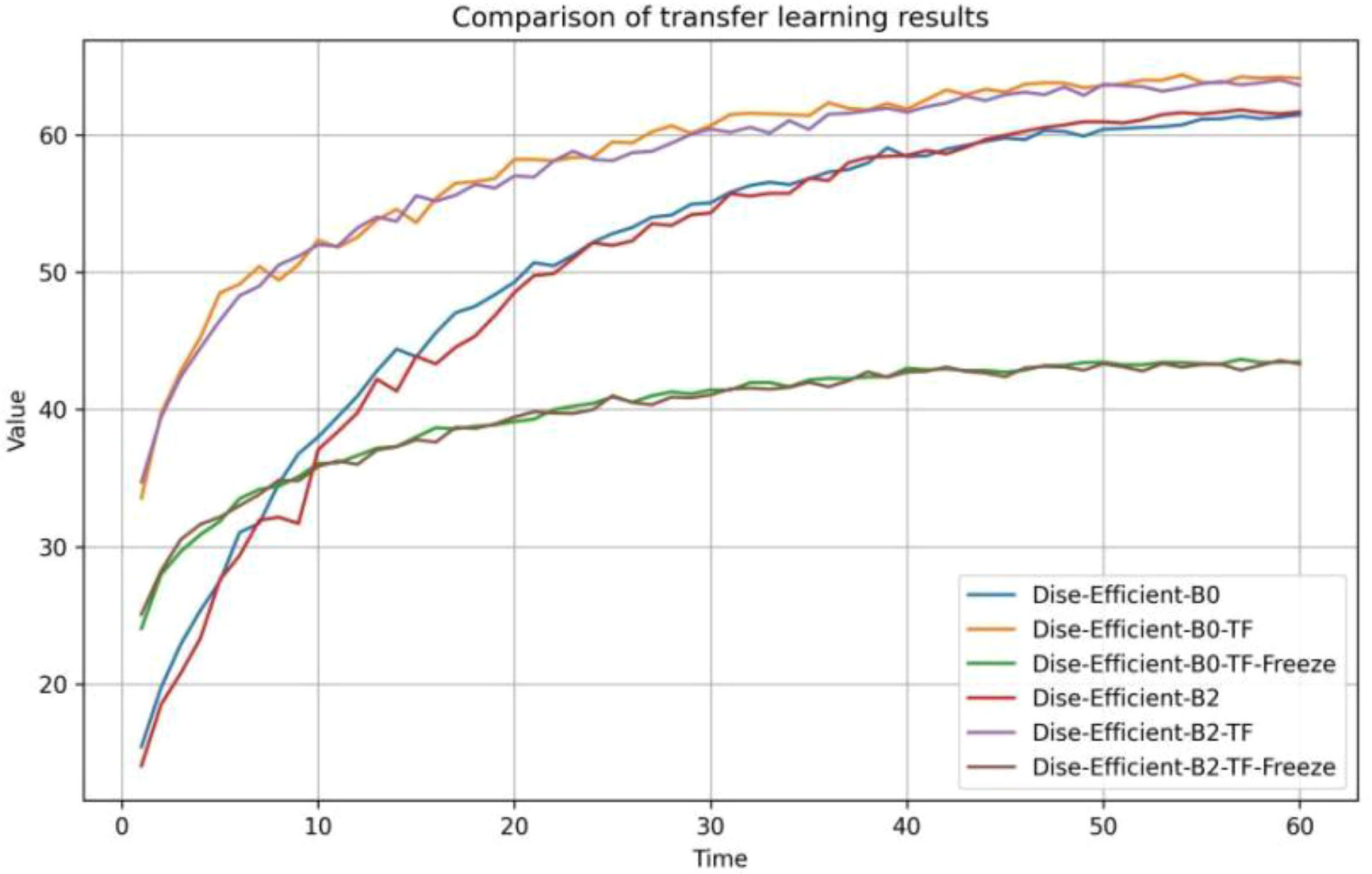
Comparison of the accuracy of the transfer learning process.

Based on the experimental design in **Table 5**, the accuracy of the model during the transfer learning process on the IP102 dataset is depicted in **Figure 7**, and the experimental results are summarized in **Table 10**.

**Table 10:** Comparison of model accuracy.

Model	Accuracy (%)	Total time spent (h)	Model	Accuracy (%)	Total time spent (h)
B0	61.48	2.49	B2	61.84	2.73
B0-Freeze	43.67(-17.81)	**1.63**	B2-Freeze	43.58(-18.26)	1.67
B0-TF	**64.40(+2.92)**	2.50	B2-TF	64.02(+2.18)	2.73

Bold values mean the line with the best model evaluation index.

It can be seen from **Figure 7** that at the beginning of training, the transfer learning model for plant pest identification delivered a higher accuracy rate than the original model. After the model training was completed, the transfer learning model with a frozen special feature layer significantly outperformed the prototype in terms of accuracy when it comes to identifying plant pests. In other words, the transfer learning training process gave the model a much stronger ability to identify plant pests accurately.

According to **Table 10**, the transfer learning effect of B0 is superior to that of B2. When under the same transfer learning conditions, the transfer learning model obtained through B0 exhibited higher accuracy rates and faster training speeds than the one obtained through B2. Moreover, freezing the feature layer and then performing transfer learning resulted in a significant improvement of over 30% in the model’s training speed. Direct transfer learning was performed on both groups of models, leading to accuracy improvements of over 2% compared to the original model.

Therefore, in practical applications, it is desirable for the Dise-Efficient model to make more precise judgments about the types of plant pests, thereby achieving accurate pest and disease prevention. Therefore, full-parameter migration holds great importance in enhancing the accuracy of the Dise-Efficient model in identifying plant pest types.

## 5 Conclusions

This present study introduces a novel Dise-Efficient model based on previous related research, capable of identifying various types of plant diseases and pests. A series of experiments were conducted to evaluate how the number of convolutional layers, learning strategy, and convolution kernel size affect the model’s performance and how transfer learning can be applied to train the model. The following conclusions have been drawn from the experiments.

The Dise-Efficient-B0-N model achieved 99.71% accuracy in identifying plant disease types on the Plant Village plant disease dataset, with a model size of 13.3 MB. In addition, the model size decreases with fewer convolutional layers, leading to a slight reduction in accuracy. In contrast, more convolutional layers result in larger model size, but there is no obvious effect on accuracy improvements.

Also on the Plant Village plant disease dataset, implementing a cosine dynamic learning rate decay strategy during the training of the Dise-Efficient-B0-N model resulted in an accuracy rate of 99.80% in identifying plant disease types, higher than that of the B0-N model. The accuracy rate of the B0-L reached 99.81%, without any overfitting. Therefore, using a cosine dynamic learning rate decay strategy can effectively improve the accuracy of the model in identifying plant disease types.

The effect of convolution kernel size on the performance of the Dise-Efficient model on the IP102 plant pest dataset was investigated through experiments. Results indicate that the accuracy rates of the Dise-Efficient-B0 and Dise-Efficient-B2 models in identifying plant pest types on this dataset were 61.48% and 61.84%, respectively, exceeding those of other advanced models in this field. Furthermore, the experimental results suggest that replacing small convolution kernels with larger ones in the Residual layer of the Dise-Efficient model is effective in improving the model’s accuracy in identifying plant pest types.

The results obtained through the transfer learning experiment conducted on the IP102 plant pest dataset demonstrate that freezing the feature layer of the pre-trained model during transfer learning training increases the model training speed by more than 30%, which, however, comes at the cost of greatly reduced accuracy. Conversely, performing full-parameter transfer learning training on the pre-trained model keeps the model training speed unchanged while increasing the accuracy of the obtained model by more than 2%. These findings demonstrate the strong transfer learning ability of the Dise-Efficient model and suggest full-parameter transfer learning as an effective approach to improve the model’s accuracy in identifying plant pest types.

In summary, our proposed Dise-Efficient model can effectively identify various types of plant diseases and pests, thereby contributing to preventing them in agricultural production. The baseline model Dise-Efficient-B0 exhibits the most comprehensive performance and boasts a compact size of only 13.3MB, making it ready for deployment in almost all kinds of lightweight mobile device applications. Specifically, the Dise-Efficient-B0 model achieves an accuracy rate of 99.80% for plant disease identification on the Plant Village dataset and an accuracy rate of 64.40% for plant pest type identification on the IP102 pest dataset after full-parameter transfer learning training. Consequently, it is anticipated that the Dise-Efficient-B0 model will be one of the top-performing models for plant disease and pest identification.

## Data availability statement

The original contributions presented in the study are included in the article/supplementary material. Further inquiries can be directed to the corresponding author.

## Author contributions

HG: Paper writing and deep learning algorithm research. CF: Design of Dise-Efficient Plant Pest Type Recognition Model. GZ: Design of paper experiments. KL: Collation and analysis of the experimental data of the paper. PW and ZZ: Research and analysis of related work for the thesis. All authors contributed to the article and approved the submitted version.

## References

[B1] Ahmad A. Saraswat D. El Gamal A. (2022). A survey on using deep learning techniques for plant disease diagnosis and recommendations for development of appropriate tools. Smart Agric. Technol. 3, 100083. doi: 10.1016/j.atech.2022.100083

[B2] Barragán-Fonseca K. Y. Nurfikari A. Van De Zande E. M. Wantulla M. Van Loon J. J. De Boer W.. (2022). Insect frass and exuviae to promote plant growth and health. Trends Plant Sci. 27 (7), 646–654. 35248491 10.1016/j.tplants.2022.01.007

[B3] Bedi P. Gole P. (2021). Plant disease detection using hybrid model based on convolutional autoencoder and convolutional neural network. Artif. Intell. Agric. 5, 90–101. doi: 10.1016/j.aiia.2021.05.002

[B4] Behmann J. Mahlein A. K. Rumpf T. Römer C. Plümer L. (2015). A review of advanced machine learning methods for the detection of biotic stress in precision crop protection. Precis. Agric. 16, 239–260. doi: 10.1007/s11119-014-9372-7

[B5] Canassa F. Esteca F. C. Moral R. A. Meyling N. V. Klingen I. Delalibera I. (2020). Root inoculation of strawberry with the entomopathogenic fungi metarhizium robertsii and beauveria bassiana reduces incidence of the twospotted spider mite and selected insect pests and plant diseases in the field. J. Pest Sci. 93 (1), 261–274. doi: 10.1007/s10340-019-01147-z

[B6] Chen J. Zhang D. Suzauddola M. Nanehkaran Y. A. Sun Y. (2021). Identification of plant disease images *via* a squeeze-and-excitation MobileNet model and twice transfer learning. IET Image Process. 15 (5), 1115–1127. doi: 10.1049/ipr2.12090

[B7] Elnahal A. S. El-Saadony M. T. Saad A. M. Desoky E. S. M. El-Tahan A. M. Rady M. M.. (2022). The use of microbial inoculants for biological control, plant growth promotion, and sustainable agriculture: a review. Eur. J. Plant Pathol. 162 (4), 759–792. doi: 10.1007/s10658-021-02393-7

[B8] Fuentes A. Yoon S. Kim S. C. Park D. S. (2019). A robust deep-Learning-Based detector for real-time tomato plant diseases and pests recognition. Sensors Agric. 1, 17, 153. doi: https://doi.org/10.3390/s17092022 PMC5620500 28869539

[B9] Ganatra N. Patel A. (2020). Performance analysis of fine-tuned convolutional neural network models for plant disease classification. Int. J. Control Automation 13 (3), 293–305.

[B10] Gokulnath B. V. (2021). Identifying and classifying plant disease using resilient LF-CNN. Ecol. Inf. 63, 101283. doi: 10.1016/j.ecoinf.2021.101283

[B11] He K. Zhang X. Ren S. Sun J. (2016). “Deep residual learning for image recognition,” in Proceedings of the IEEE conference on computer vision and pattern recognition 770–778.

[B12] He T. Zhang Z. Zhang H. Zhang Z. Xie J. Li M. (2019). “Bag of tricks for image classification with convolutional neural networks,” in Proceedings of the IEEE/ CVF conference on computer vision and pattern recognition. 558–567.

[B13] Howard A. Sandler M. Chu G. Chen L. C. Chen B. Tan M..(2019). “Searching for mobilenetv3,” in Proceedings of the IEEE/CVF international conference on computer vision. 1314–1324.

[B14] Hu J. Shen L. Sun G. (2018). “Squeeze-and-excitation networks,” in Proceedings of the IEEE conference on computer vision and pattern recognition. 7132–7141.

[B15] Hughes D. Salathé M. (2015). An open access repository of images on plant health to enable the development of mobile disease diagnostics. arXiv preprint arXiv:1511.08060. doi: https://doi.org/10.48550/arXiv.1511.08060

[B16] Kamal K. C. Yin Z. Wu M. Wu Z. (2019). Depthwise separable convolution architectures for plant disease classification. Comput. Electron. Agric. 165, 104948. doi: 10.1016/j.compag.2019.104948

[B17] Krizhevsky A. Sutskever I. Hinton G. E. (2017). Imagenet classification with deep convolutional neural networks. Commun. ACM 60 (6), 84–90. doi: 10.1145/3065386

[B18] Kumar V. Arora H. Sisodia J. (2020). Resnet-based approach for detection and classification of plant leaf diseases. In 2020 international conference on electronics and sustainable communication systems (ICESC). (IEEE), 495–502.

[B19] Lin S. Xiu Y. Kong J. Yang C. Zhao C. (2023). An effective pyramid neural network based on graph-related attentions structure for fine-grained disease and pest identification in intelligent agriculture. Agriculture 13 (3), 567. doi: 10.3390/agriculture13030567

[B20] Liu J. Wang X. (2021). Plant diseases and pests detection based on deep learning: a review. Plant Methods 17, 1–18. doi: 10.1186/s13007-021-00722-9 33627131 PMC7903739

[B21] Liu Y. Zhang X. Gao Y. Qu T. Shi Y. (2022). Improved CNN method for crop pest identification based on transfer learning. Comp. Intelligence Neurosci. 2016. 10.1155/2022/9709648 PMC8942633 35341164

[B22] Loshchilov I. Hutter F. (2017). Decoupled weight decay regularization. arXiv preprint arXiv 05101. doi: https://doi.org/10.48550/arXiv.1711.05101

[B23] Mohanty S. P. Hughes D. P. Salathé M. (2016). Using deep learning for image-based plant disease detection. Front. Plant Sci. 7, 1419. doi: 10.3389/fpls.2016.01419 27713752 PMC5032846

[B24] Nanni L. Maguolo G. Pancino F. (2020). Insect pest image detection and recognition based on bio-inspired methods. Ecol. Inf. 57, 101089. doi: 10.1016/j.ecoinf.2020.101089

[B25] Nurfauzi A. H. Azhar Y. Chandranegara D. R. (2023). Penerapan model EfficientNetV2-B0 pada baseline IP102 dataset untuk menyelesaikan masalah klasifikasi hama serangga. Jurnal Repositor 5 (3), 805–814. doi: https://doi.org/10.22219/repositor.v5i3.1583

[B26] Ren F. Liu W. Wu G. (2019). Feature reuse residual networks for insect pest recognition. IEEE Access 7, 122758–122768. doi: 10.1109/ACCESS.2019.2938194

[B27] Satorras V. G. Estrach J. B. (2018). “Few-shot learning with graph neural networks,” in International conference on learning representations.

[B28] Sehrawat A. Sindhu S. S. Glick B. R. (2022). Hydrogen cyanide production by soil bacteria: biological control of pests and promotion of plant growth in sustainable agriculture. Pedosphere 32 (1), 15–38. doi: 10.1016/S1002-0160(21)60058-9

[B29] Sladojevic S. Arsenovic M. Anderla A. Culibrk D. Stefanovic D. (2016). Deep neural networks based recognition of plant diseases by leaf image classification. Comp. Intelligence Neurosci. 2016. 10.1155/2016/3289801 PMC4934169 27418923

[B30] Szegedy C. Liu W. Jia Y. Sermanet P. Reed S. Anguelov D.. (2015). “Going deeper with convolutions,” in Proceedings of the IEEE conference on computer vision and pattern recognition. 1–9.

[B31] Tan M. Le Q. (2019). “Efficientnet: rethinking model scaling for convolutional neural networks,” in International conference on machine learning. (PMLR), 6105–6114.

[B32] Tan M. Le Q. (2021). “Efficientnetv2: smaller models and faster training,”. in International conference on machine learning. (PMLR), 10096–10106.

[B33] Thenmozhi K. Reddy U. S. (2019). Crop pest classification based on deep convolutional neural network and transfer learning. Comput. Electron. Agric. 164, 104906. doi: 10.1016/j.compag.2019.104906

[B34] Wani J. A. Sharma S. Muzamil M. Ahmed S. Sharma S. Singh S. (2022). Machine learning and deep learning based computational techniques in automatic agricultural diseases detection: methodologies, applications, and challenges. Arch. Comput. Methods Eng. 29 (1), 641–677. doi: 10.1007/s11831-021-09588-5

[B35] Weiss K. Khoshgoftaar T. M. Wang D. (2016). A survey of transfer learning. J. Big Data 3 (1), 1–40. doi: 10.1186/s40537-016-0043-6

[B36] Wu X. Zhan C. Lai Y. K. Cheng M. M. Yang J. (2019). “Ip102: a large-scale baseline dataset for insect pest recognition,” in Proceedings of the IEEE/CVF conference on computer vision and pattern recognition. 8787–8796.

[B37] Zhu Z. Zhang D. Li L. Li K. Qi J. Wang W.. (2023). Knowledge-guided multi-granularity GCN for ABSA. Inf. Process. Manage. 60 (2), 103223. doi: 10.1016/j.ipm.2022.103223

